# Autonomy of the child in the South African context: is a 12 year old of sufficient maturity to consent to medical treatment?

**DOI:** 10.1186/s12910-016-0150-0

**Published:** 2016-11-02

**Authors:** Wandile Ganya, Sharon Kling, Keymanthri Moodley

**Affiliations:** Centre for Medical Ethics and Law, Department of Medicine, Faculty of Medicine and Health Sciences, Stellenbosch University, Tygerberg Campus, Cape Town, South Africa

**Keywords:** Autonomy, Consent, Child, Person, Sufficient maturity

## Abstract

**Background:**

A child is a developing person with evolving capacities that include autonomy, mental (decisional) capacity and capacity to assume responsibility. Hence, children are entitled to participatory (autonomy) rights in South Africa as observed in the Children’s Act 38 of 2005. According to section 129 of the Act a child may consent to his or her own medical treatment provided that he or she is over the age of 12 years and is of sufficient maturity and decisional capacity to understand the various implications of the treatment including the risks and benefits thereof. However, the Act does not provide a definition for what qualifies as ‘sufficient maturity’ nor does it stipulate how health professionals ought to assess the decisional capacity of a child. In addition, South Africa is a culturally diverse country. The Western liberal notion of autonomy may not necessarily find equal prominence in the mores of people with a different worldview. Hence we demonstrate a few salient comparisons between legal liberal moral theory and African communitarianism as pertinent to the autonomy of the child.

**Discussion:**

Children are rights-holders by virtue of their humanity. Their dignity as individual human persons affords them the entitlement to human rights as contemplated under the Constitution of the Republic of South Africa. However, contrary to the traditional Western notion of individual autonomous persons African societies hold a communalistic notion of person hence there is less regard for individual autonomy and rights with more emphasis on the communal good and maintaining the continuity of relationships and interdependencies shared within a community. A child considered in this view is not regarded as a full person. This implies that decisions concerning the child, including consent to medical treatment are discussed and determined by the community to which the child belongs. Lastly, in this article, we draw on the notion of capacity for responsibility to produce a pragmatic definition of sufficient maturity.

**Conclusion:**

It seems reasonable to suggest a move away from a general legal age of consent for medical treatment toward more individualised, context-specific approaches in determining the maturity of a child patient to consent to medical treatment. Perhaps, decision-making with respect to consent to the medical treatment of a child belonging to a traditional African community where the notion of a person is embedded in communitarianism ought to involve the child’s parents/guardians/caregivers where possible provided that the best interests of the child are awarded priority.

## Background

The concept of the autonomy of a child in the context of healthcare is both complex and challenging globally. In South Africa the controversy surrounding children and autonomy has come into sharp focus since the promulgation of the Children’s Act 38 of 2005 (hereinafter referred to as “the Children’s Act” or “the Act”). Much of the debate revolves around the concept of maturity and the child’s developing capacity to consent.

The process of development generally concerns progressive advances from one state, usually primitive or simple, toward another, usually more complex or advanced. Where this process typically terminates is what is colloquially (and formally) understood as maturity. There are various dimensions of maturity including emotional, biological, cognitive and social. However, for the sake of firmer pertinence to our research question we concern ourselves herein with cognitive and social maturity as they bear directly on Western liberalism and African communitarianism, the former often emphasising rationality and individualism and the latter; sociality of persons albeit not refuting the significance of other dimensions of maturity such as the emotionality of the deciding subject in decision-making. We can thus conceive of a child as a developing *person* with evolving capacities like autonomy, mental (decisional) capacity and capacity to assume responsibility. Notwithstanding possession of capacities, we must first plainly conceive of a child as a *person*; a human being. Although this plain conception of a child is indeed attractive as it admits no prejudice toward children as rights-holders, a fuller and more adequate definition is required to define when a subject becomes a person^1^ and at what age we should consider a person no longer to be regarded as a child but rather an adult [1].

The Constitution of the Republic of South Africa (hereinafter referred to as “the Constitution”) aligns itself with the African Charter on the Rights and Welfare of the Child (ACRWC) and the United Nations Convention on the Rights of the Child (UNCRC) [2, 3] with regard to exalting children as independent legal actors – as stipulated in the Act^2^, and in defining which persons are entitled to provisions entailed therein. It provides that a child is a *person* under the age of 18 years^3^ [2, 4]. It also follows that a person is considered to have attained majority at this age. Furthermore, under the Children’s Act, a child is considered a rights-holder, not merely a property or extension of her parents or an object of adult concern [2, 5]. Children are indeed persons with an evolving capacity for individual autonomy [6] hence deserve the right to express their views freely in matters affecting them^4^. The relevant sections in the Children’s Act attend to the various jurisdictions of a child but of particular interest to us is section 129 of the Children’s Act which pertains to the consent of children to medical treatment. Section 129 expressly dictates the prerequisites for the medical treatment of a child and stipulates as follows:

‘(2) A child may consent to his or her own medical treatment or to the medical treatment of his or her child if-

- the child is over the age of 12 years; and
- the child is of sufficient maturity and has the mental capacity to understand the benefits, risks, social and other implications of the treatment.’ [4]

In the past, when the Child Care Act 75 of 1983 was still in effect, only children above the age of 14 years could consent to medical treatment [7]. What necessitated law reform was a realisation of a number of shortcomings experienced with the Child Care Act^5^ and a need to fully acknowledge children as rights-holders. A lower threshold for age of consent was thus seen as a means to promote access to health services, promote participation of children in health decisions affecting them in accordance with international trends [7, 8]. Over the years there has been mounting empirical evidence suggesting lowering age thresholds for decisional capacity in children. For example it has been demonstrated that children below 12 years can make well considered decisions [9] and that children as young as nine years old can understand issues pertinent to decision making in clinical trials [10] however the statutory age of consent to medical treatment as stipulated in various countries appears arbitrary as it varies from 12 to 19 years [11].

A child contemplated under the Children’s Act must satisfy two requirements before accessing medical treatment on his or her own, that is, without parental, guardian, or care-giver’s consent being required. The first requirement is that the child must have reached 12 years of age to consent. The second requirement is that the child must have ‘sufficient maturity’ and decisional capacity to understand the ‘benefits, risks, social and other implications of the treatment.’^6^ [12] However, there are a few deficiencies in this section of the Act with regard to definitions, regulations and sufficient descriptions [8]. Firstly, the Act does not provide a definition regarding what ought to be considered medical treatment. Hence, for the purposes of this article, we define medical treatment as a non-invasive intervention usually in the form of a drug^7^. Secondly, the Act also does not provide a definition of sufficient maturity. Hence, we will comprehend that the Act infers by ‘sufficient maturity’ a degree of cognitive development that affords a child the kind of engagement necessary in decision-making comparable to that of fully developed persons, namely*,* adults^8^. We will provide an alternative rendition of ‘sufficient maturity’ in the course of this article. Thirdly, there is no provision in the Act specifying how the health practitioner ought to assess a child’s decisional capacity. This is compounded by the fact that there currently is no standard objective tool for assessing the decisional capacity of children [9].

Moreover, considering that South Africa is a culturally diverse country [5] another concern with regard to the implementation of the Act involves the potential consequence of conferring (autonomy) rights on children without commensurate responsibilities to their community^9^. For ‘[i]n the African context, for example, individual autonomy is of smaller status than the pursuit of the communal good’^10^ [5]. In view of this, it appears the conferring of autonomy rights on children without cementing their reciprocal duties erodes interdependent relations between the child and his or her community [13, 14].

Our research question may be posited as follows: given the newest developments in child law as regards the conception of the child and his or her participation in society, how may appeals to different moral theories (African communitarianism and Western liberalism) aid in finding better and alternative means of determining *how* and *by whom* decisions about medical treatment of the child should be made? Perhaps there has not been a time better suited to address questions of this nature than today given the near-universal advocacy for children’s rights and the resurgence of activism and scholarly criticism against old hegemonic conceptions such as the status of children in civil society, person and personhood and so forth.

In advancing forth our argument we first assert children as rights-holders, give an overview of the doctrine of informed consent and the principle of respect for individual autonomy and the legal conception of a person in the setting of the Constitution; discuss African communitarianism with regard to its notion of a person and personhood and the child and the implications thereof in the consent of children to medical treatment. And in pursuit of a case-specific definition for sufficient maturity we appeal to the notion of capacity for responsibility. Lastly, in view of both legal liberal and African communitarian moral vantages we conclude by giving due attention to the enquiry whether a 12 year old is of sufficient maturity to consent to medical treatment with the conviction that no moral theory should be assigned an absolute (moral) value *a priori*, that is, antecedent to the context within which it is to be observed and/or contemplated.

## Discussion

### Asserting children as rights-holders

As a point of departure, a child is a developing person. When he or she obtains decisional capacity of such degree that affords him or her the kind of engagement necessary in decision-making comparable to that of fully developed persons, *viz.* adults, we will comprehend that as what is inferred by the Act as ‘sufficient maturity’. It appears to follow from this that a child with sufficient maturity ought to be equally afforded autonomy rights in decision-making, including medical treatment as is the case in adults. For ‘[c]onferring rights on children is viewed as ‘*recognising their moral equality with adults, thereby underscoring the moral worth of all human beings, irrespective of their situation*.’ (emphasis added) [12], and by autonomy rights we understand broadly those entitlements persons have which allow them the freedom of involvement in matters affecting them as members of civil society, be they public or private (also referred to herein as participatory rights); different perhaps to rights in general which are often conceived as entitlements persons have plainly by virtue of being persons . Having said that, do these rights also extend to those children who do not possess sufficient maturity and/or decisional capacity? The Act is unambiguous on this issue. Where a child is judged to lack sufficient maturity and decisional capacity to understand the benefits, risks, social and other implications of the treatment the authorisation of his or her consent devolves on the parent, guardian or caregiver. However, this question highlights a central problem in many rights theories as to what we mean by the notion of rights and who qualifies to be a rights-holder [15]? For if the conferring of participatory rights is contingent on possession of certain dispositions or traits such as capacity, degree of maturity, age, condition of dependency and so on as some commentators might argue then holding such rights indeed becomes exclusionary and further, fails dismally in serving the very groups it was purposed to protect [12, 15]. Hence, we maintain: if we are to truly recognise the moral equality of children with adults we ought to grant that capacity of whatever kind need not be the arbitrating principle on the conferring of rights on children.

Admissibly, as Mosikatsana observes, ‘[t]he difficulty with granting children rights is that their physical, emotional, and intellectual immaturity cause dependence on adults to assist children in exercising those rights’ [13], but, as O Neill writes (as cited by Mosikatsana) the fact that children ‘cannot claim their rights for themselves…is no reason for denying them rights. Rather it is reason for setting up institutions that can monitor those who have children in their charge and intervene to enforce rights.’ [13] Therefore, as convenient a notion as sufficient maturity and decisional capacity may appear, they do confine our discourse on the rights of the child to the exclusion of others and their claims.

Moreover, children have moral status (or moral worth) plainly by virtue of being humans or persons (these terms are used interchangeably in this paper). It would indeed appear morally unsound, let alone ‘morally monstrous’ [16], for one to argue that children have lower moral status compared to adults as it also appears unlikely that one can indeed justify it with sound moral reasoning. It is rather best assuming a value theory that does not in any manner legitimise preferences to the acquisition of certain capacities in the development of persons [17] in order for us to arrive upon the conclusion of equal rights and moral status of all persons plainly by virtue of their humanity. And by humanity we broadly refer to the totality of universal potentialities, qualities and dispositions which both constitute and distinguish us as persons, whatever these may entail. Here the conferring of rights is then premised solely on the notion of humanity and not on some other contingent condition. (This argument equally applies to the entities enumerated in the following developmental continuum: ‘blastocyst, zygote, embryo, foetus, neonate, baby, infant, child, minor, adolescent, adult’ [16].) This attribution of rights founded plainly on the notion of humanity is also apparent in the preamble of the UNCRC which recognises the ‘inherent dignity and…equal… rights of all members of the human family’ [12, 18]. It follows from this that children are indeed rights-holders for the same reasons we recognise in adults (that is, their humanity) and thus should be afforded equal rights as adults including participatory rights.

However, to exercise participatory rights requires autonomy – a capacity that is acquired over time through the process of development. It is obvious that certain age-groups will lack this capacity and thus may not have the commensurate wherewithal to exercise participatory rights in decision making [19]. Although there seems little contention to assert this, it need not necessarily follow that persons judged deficient of such capacity be stripped of that right completely as the argument herein advanced is that the affordment of participatory rights should not be predicated on the basis of capacity to exercise a right but rather on the existence of fundamental human interests that deserve protection from prejudicial forces.

In contending the notion of capacity in the setting of the “rights talk” Federle writes:

Children clearly have been disadvantaged by a rights theory premised upon capacity. The incapacities of children and their concomitant need to be protected from themselves and others permit the state to restrict the activities of children in ways that would be impermissible in the case of adults. Furthermore, these incompetencies suggest that the rights children do have are somehow different, less fundamental, and more easily overridden by paternalistic concerns for the safety and well-being of children. Consequently, the courts have authorized significant restrictions on the liberty interests of children as legitimate protective measures. Nevertheless, our laws may subject children to selective and discriminatory laws with concomitantly greater restrictions on their liberty than would be sanctioned in the case of adults. [15]

It is for these reasons that we argue that children are rights-holders regardless of whether or not they possess the wherewithal to exercise these rights.

### The doctrine of informed consent

The doctrine of informed consent holds that persons are their own sovereign and should thus be allowed to make the final decision on affairs concerning them providing that the elements required for informed consent (or informed refusal) [20] have been satisfied. These elements include:

1. Competence;
2. Disclosure of information;
3. Understanding and appreciation of information disclosed;
4. Voluntariness in decision-making;
5. Ability to express a choice [5].

In view of the above it may safely be declared that informed consent has occurred when a competent person has received a thorough disclosure, understands and appreciates the disclosure, acts voluntarily, and consents to the intervention [19]. We briefly elaborate on these in the following accounts.

Competence^11^ simply refers to the ability to perform a task [20]. It is task and context-specific and changes over time. By convention, age and decisional capacity are thought to be the chief elements that constitute competence. Albeit several competence assessment tools for children have been devised by various authors e.g. Hopkin’s Competency Test, Competency Questionnaire-Child Psychiatric and the Competency Questionnaire-Pediatric Outpatient Modified Version, there currently exists no standard objective tool to assess a child’s competence to consent to medical treatment [9, 10]. This inclines assessors of competence (health practitioners) to make judgements based on subjective assessments. A patient’s competence is influenced by their experience with a medical condition, hospitalisation, family relationships and social roles and development [21].

Furthermore, ‘[i]t is a legal obligation for health practitioners to disclose relevant information to their patients regarding:

1. The patient’s health condition (except when disclosure of information would be contrary to the patient’s best interest)
2. Available diagnostic and treatment options
3. Risks, benefits, costs and consequences attached with each option
4. The option of non-treatment, that is, informed refusal and its implications.’ [5]

The patient should also attach significance to the information disclosed.

‘The process of consent should also be conducted in a language that the patient understands and in a manner that considers the patient’s level of literacy. This is especially so with children.’ [5].

In addition, for informed consent to be valid it must be voluntary, that is, the patient must not be influenced by other individuals either by coercion, persuasion or manipulation [5, 19].

Lastly the patient’s choice to treatment or non-treatment may be expressed orally, in writing or may be implied, that is, tacit consent [19, 22].

### Capacity for responsibility

A deciding subject, in this instance a child, ought not to only consider given choices but also accept the prospective responsibilities involved. And to ‘*accept responsibility means to be able to be held accountable for whatever decisions are taken, on the basis of the assumption that reasons can be provided, that they have been thought through, and even though they might be fallible.*’ [23]. That is, a deciding child must also have the capacity for responsibility for that particular choice decided upon, whatever this may entail. Capacity for responsibility therefore refers to a deciding subject’s ability to deal with the likely outcomes of his or her decision.

Whilst we grant that a person need not possess capacity of any kind to have moral status and constitutional rights (human dignity, privacy, freedom), as we established above, we argue that a deciding subject *must* then necessarily possess or be facilitated insofar as it is practically possible to possess the commensurate wherewithal for responsibility to account for that particular choice decided upon. In view of this, we arrive at our ultimate definition of ‘sufficient maturity’:

*A child has sufficient maturity to consent to medical treatment insofar as he or she can independently demonstrate (or be facilitated either by aids or a helper as far as it is practically possible in that given setting to possess) the commensurate wherewithal required to assume responsibility for that specific decision.*

To clarify this definition, let us make an example: a child patient is newly diagnosed with type I diabetes mellitus and it is required that she consents to using insulin injections as her treatment. To determine whether she has sufficient maturity to consent to using insulin injections the health practitioner must consider, among other factors, whether the child would be able to take the chronic medication as frequently as prescribed. A child who has previous experience with a chronic illness like asthma may be presumed to already possess the capacity to assume the responsibility of taking chronic medication. Those children whom it is believed cannot demonstrate the forgoing capacity in order to assume responsibility can be facilitated to attain this capacity. In the case where a child patient refuses treatment we advise that recourse be made to the best interest principle. A child (or adult) who fails this definition of sufficient maturity may be considered incompetent to make a decision.

### The Constitution on autonomy and the legal conception of a person

Human dignity is expressly enumerated in the Bill of Rights Chapter of the Constitution as a human right that deserves respect and protection. It is a foundational value that ‘informs the interpretation of other specific rights’ [24]. Albeit some authors, such as Jordaan [24], claim that one of the fundamental elements of human dignity include the capacity for *autonomy* whether understood as free-will or rational deliberation [25], we maintain throughout this paper that human dignity in general denotes a universal, and objective value *inherent* to all human *persons* notwithstanding capacity*.*

The notion of *autonomy* is derived from the Greek expressions: ‘*autos*’ – self, and ‘*nomos*’ – law, referring to a self-legislating agent [19, 24, 25]. Autonomy is a constitutional value defined by the Courts as ‘the ability to regulate one’s own affairs, even to one’s own detriment’ [24]. Implicit in this juridical definition is the acknowledgement of autonomy as a developmental phenomenon. This is inferred by the term “ability” implying that autonomy is an evolving capacity that is, *acquired* in the process of human development. According to the provisions of the UNCRC and the ACWRC, a child has autonomy rights. The Children’s Act first defines a child as a *person* below the age of 18 years and further specifies in section 129 which children can fully exercise autonomy rights in the setting of consent to medical treatment (as dealt with above) [4] It is plain from the forgoing definition of a child that rights are ascribable only to *persons* not things^12^. According to Black’s Law dictionary a natural *person* considered in juridical contexts is a human being; a legal entity with rights and duties that deserve protection and respect [26]. However, it still remains unclear as to what is truly meant by the notion of *person* or *human being*; what potentialities, qualities and dispositions declare us as persons and thereby entitle us to constitutional rights (e.g. autonomy rights), and duties in general. We acknowledge the import of such a definition as a desideratum not only in juridical but also in philosophico-ethical contexts with regard to moral status and abortion.

### African communitarianism on autonomy, the conception of a person and the consent of a child to medical treatment

‘Amidst gathering talk of human rights and civil society, of the celebration of autochthony and authenticity, the version of an African Renaissance arises to counter the rampant excesses of European modes of being-in-the world’ [27]

Communitarianism is a moral theory concerned with the pursuit of the communal good. It expressly repudiates individual autonomy (and liberal moral theory) and exalts community. In this theory, individual rights become docile whilst duties owed by a member to his or her community are held to be of great import, and communal values such as mutual reciprocity, collective loyalties and solidarity are endorsed [19, 28]. The consideration of a *person* has always been at the centre of consternation in this moral theory. The problem can be stated as follows: is a person wholly embedded in a communal matrix of interrelations and interdependencies without the concession of individual autonomy as radical communitarians insist or does one retain his or her individual merits like autonomy within a community as moderate communitarians argue?^13^ [19, 28].

African societies generally uphold communal values (African communitarianism), of those, the highest weight is assigned to relationships shared within a community [19, 28, 29], and to human life (vitality). Thus, a *person* has the duty to preserve the continuity of such relationships by pursuing the communal good, whatever this may entail. In traditional African thought a person exists as an extended entity embedded within a communal matrix of interrelations and interdependencies, owing much to the relational nature of human beings. Thus, a person is regarded as an ontological and epistemological reference thereof [28, 29]. This concept of a person is no better expressed than in John Mbiti’s coinage of the African ethos:

‘I am because we are; and since we are, therefore I am’ [30]

Personhood in the African ethos is thought to be *acquired* through a process of incorporation into the community [29] and this involves executing one’s duties owed to the community. And we may add here that this requires a good measure of social maturity. Personhood in this view is something that one can indeed fail. Moreover, in this view a child is not considered as a *person* as *it* is yet to fulfil *its* duties to attain personhood [29]. This however raises an important question: How can we acknowledge the rights of children (as we asserted elsewhere) if we cannot conceive of them as full persons? To answer this we appeal to an alternative interpretation of the notion of human dignity established upon the African communitarian value for vitality [25] as opposed to autonomy and declare this as follows: a child (or being) has human dignity thereby human rights insofar as he or she has vitality^14^.

In truth, however, African communitarianism^15^ is premised upon a duty-based system; not naturally perceived nor experienced as being oppressive to the individual since the individual himself or herself realises his or her interests as being consonant with the pursuit of the communal good and sees nothing else outside this [31]. He or she therefore finds little sense in “going on” about individual rights that seemingly conflict with the harmony of interrelations and interdependencies shared within the very community whence he or she derives self-worth and security with regard to individual welfare.

In healthcare where informed consent is a necessary ethical and legal requirement to solicit from a patient before performing an indicated medical intervention a patient from an African communitarian society may often wish to consult with his or her community to make a decision [32, 33]. This derives from the fact that in African communitarian societies the best interests of all persons, not only the child, are determined by the community based on the communal value-system. Hence important decisions are arrived upon through collective discussions, often in the presence of elders from the community since their wisdom is highly regarded concerning (moral) decision-making to guard the interest of the community. Where consent to the medical treatment of a child (or person) is concerned it is likely that the community from which the child belongs will collectively decide on this. It appears therefore that the African value system is indeed in conflict with the law which permits a child 12 years or older to make an autonomous decision regarding his/her medical treatment.

## Conclusion

It seems reasonable to suggest that we move away from a general age of consent toward more individualised, context-specific approaches in determining the maturity of a child patient to consent to medical treatment. Conferring rights upon children based on capacity (autonomy rights and decisional capacity in this instance) alone may be myopic at best. Hence, we subscribe to the minimal notion that where a child is able to express his or her will based on an established value system and rationality they ought to have their views taken seriously in decisions pertaining to their medical treatment. We also suggested an alternative definition of sufficient maturity which can be used to determine whether a child patient is indeed competent to consent to medical treatment or not without unfairly discriminating against children based on their perceived incapacities. The proposed definition places emphasis on the prospective duties that the decision-maker ought to be able to execute consequent to his or her decision.

In South Africa conflict exists between law and the African value system. In view of both legal liberal and African communitarian moral theories it is however plain that no one theory can account for how we ought to conceive of a child and his or her freedom to consent to medical treatment antecedent to the context within which the child is raised. Hence, we argue that no moral theory should be assigned an absolute (moral) value *a priori*, that is, antecedent to the context within which it is to be observed and/or contemplated and propose that in the case of a child who belongs to an African communitarian society decision-making with respect to consent to her medical treatment ought to involve the child’s community (included here are the child’s parents/guardians/caregivers) insofar as it is possible provided that the best interests of the child are awarded priority.

## Abbreviations

ACRWC: African Charter on the Rights and Welfare of the Child
UNCRC: United Nations Convention on the Rights of the Child

## Footnotes

1 Alternatively, when does personhood or childhood begin?

2 The Children’s Act 38 of 2005 is the most significant statute in South Africa entailing provisions and protections of the constitutional rights of the child. Among these rights are those pertaining to the participation of children in health treatment decisions.

3 See Article 2 of the ACRWC and Article 1 of the UNCRC. South Africa ratified the ACRWC in 2000 and the UNCRC in 1995.

4 See section 10 of the Children’s Act.

5 Some of these shortcomings included difficulties finding the caregivers or foster parents of orphans or child-headed households when required to consent to medical treatment thereby access health services.

6 This requirement derives from *the Gillick v West Norfolk and Wisbech Area Health Authority* case and is commonly referred to as the “*Gillick* competence test” [8].

7 However, we also maintain that regardless of whether an intervention is indeed medical or not recourse should always be made to the principle of respect for human dignity as enshrined in the Constitution.

8 It still remains to be proven whether such degree of maturity can indeed be clearly defined, measured and objectively validated with the support of empirical evidence.

9 The term “community” will be used broadly to also denote family as a communal unit.

10 The communal good involves preserving the continuity of communal interrelations and interdependencies.

11 In this article we use the terms *competence* and *capacity* interchangeably.

12 A calabash for example cannot conceivably have rights (or duties) insofar as any rational person cannot conceive of it a person, or rather, insofar as it is not a person notwithstanding its aesthetic, economic and social value.

13 For the sake of brevity we will not engage this enquiry any further, saving it for another occasion.

14 Vitality may be thought as a being’s ability (or potentiality thereof) to ‘exhibit a superlative degree of health, strength, growth, reproduction, creativity, vibrancy, activity, self-motion, courage and confidence, with a lack of life force being constituted by the presence of disease, weakness, decay, barrenness, destruction, lethargy, passivity, submission, insecurity and depression’ [25].

15 The terms “African communitarianism”, “African context” and “traditional African thought” are employed in the general sense where that which we denote as “African” refers to whatever it is that relates to the continent’s indigenous cultures, people and heritage. Often it is asked how can one speak broadly of an African context, thought or ethos and so given the diversity within the continent. To answer this question let us imagine for instance there being culture *p* (*p* being one of the indigenous cultures of Africa). Although culture *p* may not be a microcosm of the African’s mode of being in the world, it may be said that it shares certain commonalities with other indigenous cultures *mutatis mutandis* to permit reference to our observations in *p* as “African”, especially if one considers African cultures forming intersecting lines or partly overlapping circles; where they intersect or overlap we may speak of specific (ethnocultural) universals which one may broadly denote as African.
